# Disseminated Intravascular Coagulation Following ¹⁷⁷Lu-Dotatate Therapy in a Patient With Metastatic Midgut Neuroendocrine Tumour: A Case Report

**DOI:** 10.7759/cureus.96945

**Published:** 2025-11-16

**Authors:** Hassan Sharif, Simon McRae

**Affiliations:** 1 Haematology, Launceston General Hospital, Launceston, AUS

**Keywords:** 177lu-dotatate, disseminated intravascular coagulation (dic), peptide receptor radiation therapy (prrt), peptide receptor radiation therapy(prrt), rare side effect, small bowl neuroendocrine tumors

## Abstract

Peptide receptor radionuclide therapy (PRRT) is an expanding field with ¹⁷⁷Lu-Dotatate emerging as a treatment for midgut neuroendocrine tumours. Haematological side effects are infrequent, and the treatment is generally well tolerated. We present the case of a 70-year-old male patient who developed spontaneous bruising five weeks after his first dose of ¹⁷⁷Lu-Dotatate, with investigations consistent with disseminated intravascular coagulation (DIC). After initially requiring substantial transfusion support, the patient's DIC resolved with the commencement of corticosteroid therapy. This case suggests that DIC may be a rare subacute complication of ¹⁷⁷Lu-Dotatate, and that corticosteroids could be an effective component of management in such instances.

## Introduction

Findings from the Neuroendocrine Tumours Therapy Trial (NETTER-1) trial have significantly improved treatment options for patients with advanced, progressive, somatostatin receptor-positive midgut neuroendocrine tumours. It showed that ¹⁷⁷Lu-Dotatate therapy results in longer progression-free survival and a significantly higher response rate compared to the only alternative treatment, octreotide [1].

The NETTER-1 trial reported adverse events occurring in at least 10% of patients. Notably, disseminated intravascular coagulation (DIC) was not reported as a side effect of ¹⁷⁷Lu-Dotatate therapy [1]. In a separate study, grade 3 or 4 subacute haematological toxicity occurred in 11% of patients four to eight weeks post therapy. However, DIC was again not explicitly mentioned [2].

To the best of our knowledge, only one case report to date has described delayed onset DIC as a complication of ¹⁷⁷Lu-Dotatate therapy [3]. We present a second case of DIC as a subacute complication of ¹⁷⁷Lu-Dotatate therapy. In both cases, corticosteroids combined with transfusion support led to favourable outcomes.

## Case presentation

A 70-year-old male patient presented with spontaneous intramuscular haematomas in the right deltoid and left biceps. Six months earlier, he had been diagnosed with a metastatic small bowel neuroendocrine tumour involving mesenteric lymph nodes, liver, and bone. He did not undergo chemotherapy or surgical resection but underwent treatment with octreotide 30 mg every four weeks. Given his high disease burden and uncontrolled symptoms, he was commenced on peptide receptor radionuclide therapy (PRRT) with ¹⁷⁷Lu-Dotatate therapy five weeks before presentation (octreotide was stopped).

His past medical history also included triple-vessel coronary artery disease treated with percutaneous coronary interventions to the left anterior descending, circumflex, and right coronary arteries four years prior to presentation. In preparation for PRRT, he underwent urgent tricuspid valve replacement for severe tricuspid regurgitation secondary to carcinoid heart disease, with concurrent aortic valve replacement and patent foramen ovale closure three months prior to presentation.

He received an intravenous dose of 7.7 gigabecquerels of ¹⁷⁷Lu-Dotatate five weeks prior to presentation without immediate adverse effects. At presentation to the emergency department, there was extensive bruising on both arms, but he was otherwise systemically well, and examination was unremarkable apart from the bruising. Bilateral upper limb ultrasonography did not reveal deep vein thrombosis but confirmed a right deltoid intramuscular haematoma (39 × 8 × 16 mm) and a left biceps haematoma (47 × 23 × 33 mm). Initial blood test results are shown in Table 1. 

**Table 1:** Laboratory results eGFR: estimated glomerular filtration rate; INR: international normalised ratio; APTT: activated partial thromboplastin time; WNR: within normal range

Test Type	Patient Value	Units	Reference Range	Comments
Haemaglobin	73	g/L	130-175	Low
Neutrophils	1.5	/nL	2.0-7.5	Low
Platelets	15	/nL	150-400	Low
INR	1.7		0.9-1.2	High
Fibrinogen	0.3	g/L	2.0-4.5	Low
APTT	33	Seconds	25-35	WNR
Thrombin Time	24	Seconds	15-22	High
D-Dimer	> 32.5	mg/L FEU	<0.5	High
eGFR	>90	mL/min/1.73m²	>89	WNR
Creatinine	69	umol/L	60-115	WNR
Potassium	4.6	mmol/L	3.5-5.5	WNR
Phosphate	1.2	mmol/L	0.8-1.5	WNR
Corrected Calcium	2.11	mmol/L	2.15-2.55	Low

The patient was febrile without a clear infectious source and was empirically started on piperacillin-tazobactam. It was thought his fever may have been related to the large haematomas. A respiratory viral panel was negative. Blood cultures were negative after five days of growth. The peripheral blood smear showed marked thrombocytopenia with a few acanthocytes. CT of the chest, abdomen, and pelvis showed stable metastatic disease.

The D-dimer, platelet count, and fibrinogen levels were sufficiently deranged to meet the International Society on Thrombosis and Haemostasis (ISTH) criteria for a diagnosis of DIC. The patient required initial significant transfusion support in an attempt to keep the fibrinogen level > 1.0 g/L and platelet count > 50 x 10 9/L, receiving 22 units of cryoprecipitate, four units of packed red blood cells, and five pools of platelets, predominantly during the first three days of admission.

Based on the prior case report [3], prednisolone 37.5 mg daily was initiated on day 3 of admission. The patient’s condition improved, with significant improvement in platelet count, international normalized ratio (INR), and fibrinogen levels within 48 hours of commencement. He no longer required transfusions and was discharged on corticosteroids with local follow-up arranged.

**Figure 1:**
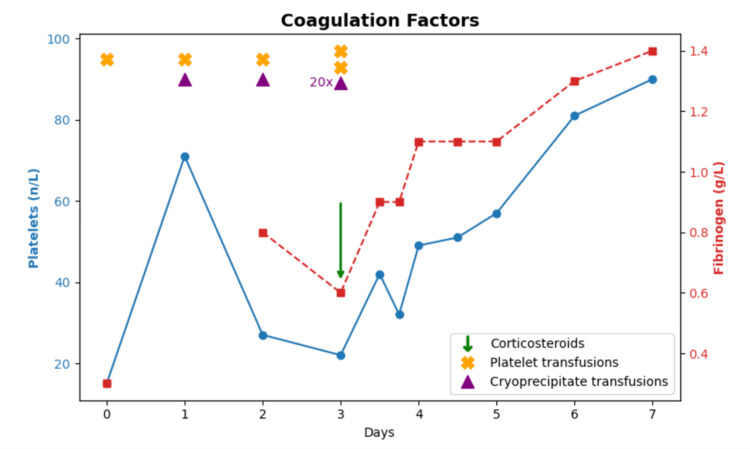
Evolution of platelet and fibrinogen levels

## Discussion

DIC has been previously reported as a complication of strontium-89 therapy for prostate cancer, although the precise mechanism remains unclear. One proposed explanation involves the release of thromboplastins during tumour cell breakdown following radiopharmaceutical administration, which subsequently triggers the coagulation cascade [4].

A similar process may underlie DIC following ¹⁷⁷Lu-Dotatate therapy, where tumour necrosis induced by the beta- and gamma-emitting isotope could theoretically activate coagulation pathways. In the previously reported case of DIC associated with ¹⁷⁷Lu-Dotatate, tumour lysis was considered a potential mechanism [3]; however, there was no biochemical evidence of tumour lysis, and it was therefore not thought to be the primary cause. In our patient, there was likewise no biochemical evidence of tumour lysis, indicating that tumour lysis was unlikely to be responsible for the observed DIC.

Alternatively, radiation itself may play a direct role. Radiation has been shown in animal studies to activate the coagulation cascade through inflammatory pathways [5]. This mechanism was also proposed in the earlier ¹⁷⁷Lu-Dotatate case [3], drawing parallels with animal data [5] and previous reports of DIC following strontium-89 therapy for prostate cancer [4]. Radiation-induced inflammation may therefore contribute to coagulopathy and could explain the favourable response to corticosteroid therapy observed in that report [3].

Although metastatic malignancy is a recognised cause of DIC, it generally follows a chronic, low-grade course that only resolves with effective treatment of the underlying cancer. In this patient, the malignancy was well controlled at the time of presentation, supporting a treatment-related or radiation-associated mechanism rather than tumour progression.

## Conclusions

There is limited literature on the role of corticosteroids in managing treatment-related DIC. Our report shows that corticosteroid administration is associated with prompt clinical improvement. DIC may represent an under-recognized subacute complication of ¹⁷⁷Lu-Dotatate therapy, and monitoring for this condition may be prudent. This case adds to the limited evidence suggesting corticosteroids may be beneficial in managing such presentations. Further research is warranted to explore the role of glucocorticoids in the management of DIC associated with radionuclide therapy.
